# Patient-derived organoid xenografts model esophageal cancer cachexia and enable assessment of anti-inflammatory drug repositioning

**DOI:** 10.1016/j.isci.2026.114638

**Published:** 2026-01-07

**Authors:** Bryan Chee-chad Lung, Alvin Ka-kiu Leung, Carissa Wing-Yan Wong, Ian Yu-hong Wong, Cheryl Chee Heng Lung, Anthony Wing-ip Lo, Josephine Mun-Yee Ko, Wei Dai, Dora Lai-wan Kwong, Simon Law, Maria Li Lung, Valen Zhuoyou Yu

**Affiliations:** 1 Department of Clinical Oncology, School of Clinical Medicine, The University of Hong Kong, Hong Kong, China; 2 Department of Surgery, School of Clinical Medicine, The University of Hong Kong, Hong Kong, China; 3 Department of Pathology, Princess Margaret Hospital, Lai Chi Kok, Hong Kong, China; 4 Division of Anatomical Pathology, Queen Mary Hospital, Pokfulam, Hong Kong, China

**Keywords:** health sciences

## Abstract

Esophageal squamous cell carcinoma (ESCC) is highly associated with cancer cachexia, a wasting syndrome lacking effective treatments. Existing animal models fail to capture key clinical and biological features of this condition. Here, we established a panel of patient-derived organoid xenograft (PDOX) models that authentically replicate the heterogeneity of ESCC-associated cachexia in immunodeficient mice. PDOX lines exhibited slow tumor growth compared with traditional ESCC xenografts. Heterogeneous cachexia phenotypes in PDOX-bearing mice, as compared with non-tumor-bearing mice, including body weight loss, reduction in adipose tissue, reduced grip strength, and elevated pro-inflammatory cytokines, were observed. Using this platform, we tested two macrophage-targeting interventions: 10 mg/kg/day rosiglitazone, a PPAR-γ agonist, and 40 mg/kg/day pexidartinib (PLX3397), a CSF1R inhibitor. Both drugs significantly attenuated cachexia-associated functional decline and systemic inflammation. Transcriptomic analyses confirmed suppression of pro-cachectic cytokine signaling. This study presents a clinically relevant platform for preclinical cachexia research and supports macrophage modulation as a potential anti-cachexia strategy.

## Highlights

• Patient-derived organoid xenografts in mice mimic esophageal cancer cachexia
• Rosiglitazone prevents tumor-induced cancer cachexia via depleting inflammatory macrophages
• PLX3397 depletes macrophages to curb systemic inflammation and blunt key inflammatory pathways
• Both rosiglitazone and PLX3997 attenuated cancer cachexia by targeting macrophages

## Graphical abstract

**Figure:**
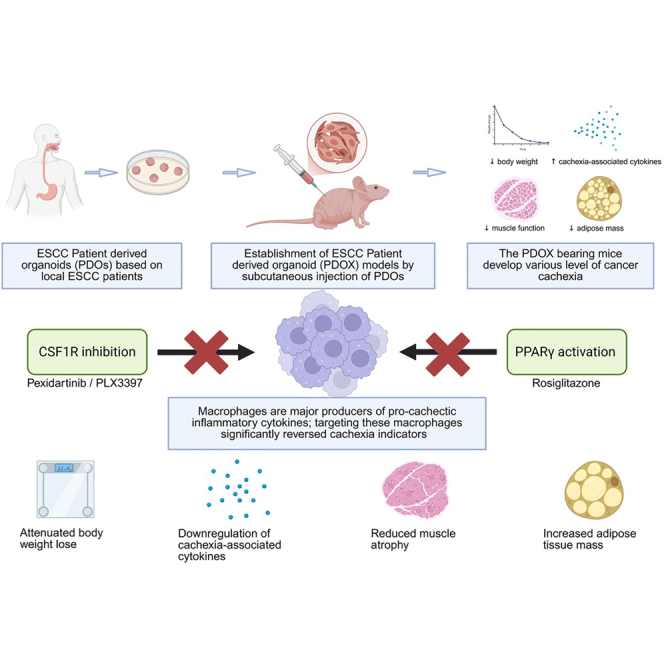

## Introduction

Esophageal squamous cell carcinoma (ESCC) is a major form of esophageal cancer, particularly prevalent in Asia. It is a deadly malignancy with a 5-year survival rate of less than 20%. ESCC is one of the cancer types with the highest incidence (60%–80%) of cancer cachexia, a severe syndrome characterized by multi-organ wasting, muscle atrophy, and metabolic dysregulation, ultimately contributing to high mortality in patients with cancer.^1^^,^^2^^,^^3^ Proinflammatory cytokines, such as interleukin-6 (IL-6), IL-1, and tumor necrosis factor-alpha (TNFα), originating from both tumor and host immune cells, play critical roles.^3^ Patients with ESCC-induced cachexia demonstrate poor tolerance to chemotherapy.^4^^,^^5^ Yet cachexia remains grossly under-addressed in patient care, with no approved pharmacologic therapies to date.^6^^,^^7^^,^^8^

Progress in understanding cachexia biology has been hampered by the scarcity of suitable preclinical models. The few existing animal models are predominantly syngeneic,^9^ but they induce rapid wasting at unrealistically high tumor burdens and fail to capture the molecular signatures of human cachexia.^9^^,^^10^ Recent studies have expanded the modeling repertoire: Liu et al. identified tumor-host metabolic interactions in *Drosophila* and murine systems, showing that tumor-derived cytokines such as Upd3/IL-6 drive hepatic gluconeogenesis via PEPCK1 and PDK3^11^; Choi et al. applied integrative metabolic profiling to dissect systemic alterations in a mouse cachexia model^12^; and Weinzierl et al. established a KPP pancreatic cancer model revealing sex-specific cachexia outcomes.^13^ While these advances highlight important insights, they also underscore the limitations of existing platforms. To date, no patient-derived xenograft (PDX) or patient-derived organoid xenograft (PDOX) models of cancer cachexia have been reported.

Recent preclinical studies have highlighted the therapeutic potential of insulin sensitizers and cytokine inhibitors—specifically those targeting IL-1, IL-6, and TNF-α—in the treatment of cancer cachexia across various tumor models, demonstrating encouraging outcomes.^14^ Increasing evidence also emphasizes the leading role of inflammatory macrophages in the pathogenesis of cachexia, as they are major producers of pro-cachectic inflammatory cytokines; consequently, targeting these macrophages represents a compelling strategy to mitigate the multi-organ wasting syndrome that defines cancer cachexia.^15^^,^^16^

To advance cancer cachexia research, a physiologically relevant preclinical model is essential. Recent work in pancreatic cancer suggested that organoid xenografts could model cachexia features, though robust wasting was not realized in their setting.^17^ We developed a panel of PDOX models from fresh ESCC biopsies. This model closely mirrors key histological and molecular features of esophageal cancer and the variable cachexia phenotypes seen in patients. It enables robust tumor-host interactions, leading to hallmark cachexia features such as weight loss and tissue wasting. Using this model, we evaluated two repurposed drugs—the FDA-approved PPAR-γ agonist rosiglitazone (RGZ) and the preclinical macrophage-targeted CSF1R inhibitor pexidartinib/PLX3397—which effectively attenuated cachexia, likely by targeting pro-inflammatory macrophages. These concise, proof-of-concept findings highlight the value of our PDOX model for mechanistic and therapeutic studies of cancer cachexia.

## Results

### Heterogeneity in PDOX growth and cachexia phenotype

**Figure 1:**
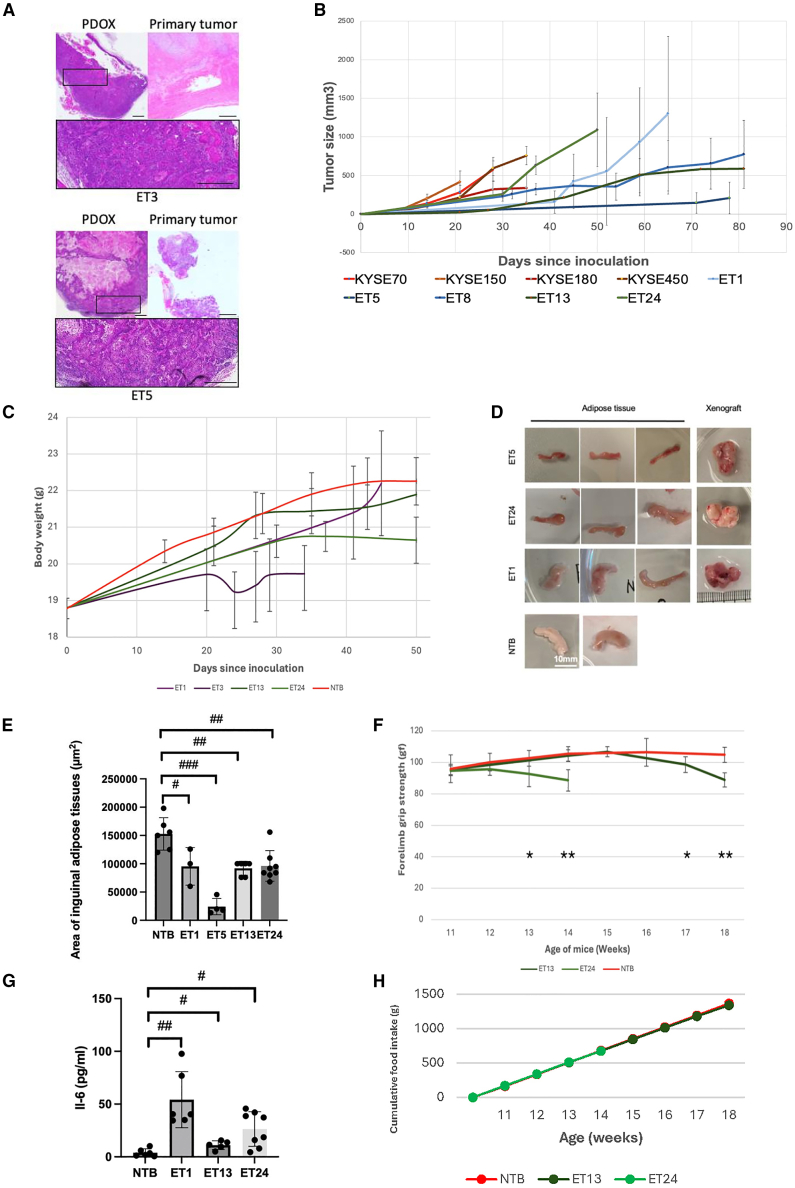
PDOX models recapitulate heterogeneous esophageal cancer cachexia phenotypes (A) Representative histopathological images (H&E staining) of ET3 and ET5 tumors in PDOX models (left) and their corresponding primary tumors (right). ET3 represents an earlier-stage tumor with moderate differentiation, whereas ET5 is a late-stage ESCC tumor with poor differentiation. Both PDOXs preserved key histopathological features of the original tumors, including keratinization nests and squamous differentiation, demonstrating faithful recapitulation of patient tumor architecture. *Scale bars, 400 μm*. (B) Tumor growth comparison between slower-growing PDOX models (ET1, ET5, ET8, ET13, ET24) and rapidly proliferating cell line-derived xenografts (CDXs; KYSE70, KYSE150, KYSE180, KYSE450). Non-tumor-bearing (NTB) mice are included as controls. (C) Body weight comparison across four PDOX lines (ET1, ET3, ET13, ET24) and NTB controls, highlighting varied cachexia onset and progression. (D) Representative images illustrating adipose tissue wasting in inguinal adipose tissues (IAT) from PDOX-bearing mice (ET1, ET5, ET24) versus NTB controls. Images were selected to illustrate the representative phenotype, excluding extreme outliers. *Scale bars, 10 mm*. (E) Quantitative analysis of inguinal adipose tissue areas across PDOX lines: NTB = 152833, 95% confidence interval (CI) [129807–175860]; ET1 = 95333, 95% CI [57712–132955]; ET5 = 24250, 95% CI [10114–38386]; ET13 = 92000, 95% CI [82083–101917]; ET24 = 961250, 95% CI [77346–114904]). (F) Forelimb grip strength assessment in PDOX-bearing mice: ET13 (mild cachexia) and ET24 (severe cachexia) compared with NTB controls. (G) Plasma IL-6 cytokine levels quantified by ELISA across PDOX-bearing mice. (NTB = 3.88, 95% CI [0.98–6.78]; ET1 = 54.13, 95% CI [32.83–75.42]; ET13 = 11.05, 95% CI [7.48–14.62]; ET24 = 26.31, 95% CI [14.31–38.38]). (H) Cumulative food intake measured per cage (6 mice per cage) in ET13 and ET24 mice, with NTB as controls.

We previously established and characterized a panel of PDOs from ESCC and esophageal adenocarcinoma patient samples; these diverse PDO cultures closely resembled patient tissue samples and exhibited varying levels of differentiation and proliferation characteristics, serving as an optimal preclinical model for drug testing^18^ (Table S1). In this study, we successfully generated a panel of 12 ESCC PDOX models, providing a robust platform for preclinical drug testing^18^ (Table S1). The established PDOXs exhibited typical histopathological features of ESCC, consistent with the original primary tumor (Figure 1A). Consistent with findings from other studies involving PDXs and PDOXs, ESCC PDOXs exhibited markedly slower *in vivo* proliferation compared with cell line-derived xenografts (CDXs). This slower growth rate allows prolonged interaction between cancer cells and the host microenvironment, facilitating the development of chronic disease features that more closely resemble human cancers—including cancer cachexia, which was not observed in any CDX-bearing mice (Figure 1B).^9^ In contrast, CDX tumors typically display a highly proliferative phenotype, with all tested models reaching a tumor volume of 150 mm^3^ within 21 days. In comparison, only 20% of PDOXs reached the same tumor size within the time frame. Most PDOX-bearing mice reached humane endpoints due to tumor burden within 1–2 months post-inoculation, without showing signs of cachexia (Figure 1B). Additionally, the slower growth rate helps reduce the immunomodulatory effects caused by the rapid expansion of CDXs upon implantation. The slower proliferation of PDOXs enabled sustained cachexia-related changes, highlighting the clinical relevance and advantage of the models in cachexia studies.

Cachexia phenotypes in PDOX-bearing mice typically emerged within 20–40 days post-inoculation, characterized primarily by progressive body weight loss (Figure 1C), though notable inter-individual variability was observed, with some mice showing modest weight gain and others exhibiting losses approaching 10% (Figure S1).This was accompanied by a marked reduction in adipose tissue, as evidenced by significantly smaller endpoint inguinal adipose tissue (IAT) areas in PDOX-bearing mice compared with non-tumor-bearing (NTB) controls (81,142 ± 15,060 μm^2^ vs. 152,833 ± 23,026 μm^2^, *p* < 0.001; Figures 1D and 1E). Additional signs of cachexia included decreased grip strength (Figure 1F) and elevated expression of cachexia-associated cytokines (Figure 1G). Importantly, these cachexia features occurred independent of changes in food intake (Figure 1H), ruling out simple anorexia and highlighting systemic inflammation-driven cachexia. These effects were observed regardless of tumor size, highlighting the heterogeneous characteristics of esophageal cancer cachexia (Figure 1D). Notably, the ET24-bearing mice developed a severe but less acute form of cachexia, providing a representative but extended window for therapeutic evaluation. These mice exhibited a significant decrease in body weight (−2.01g, *p* = 0.049) and grip strength (−17.8 gf, *p* = 0.007) compared with the NTB group on day 50 post-tumor inoculation.

### Effects of RGZ on esophageal cancer cachexia

Given accumulating evidence that activation of PPAR-γ counteracts cancer-induced wasting by suppressing inflammation and protecting muscle and adipose tissues,^19^ we selected RGZ, an FDA-approved PPAR-γ agonist,^20^ for proof-of-concept testing using our PDOX cachexia platform. Two representative PDOX lines with moderate (ET13) and severe (ET24) cachexia phenotypes were used to evaluate the therapeutic efficacy of RGZ under varying baseline cachexia severities.

**Figure 2:**
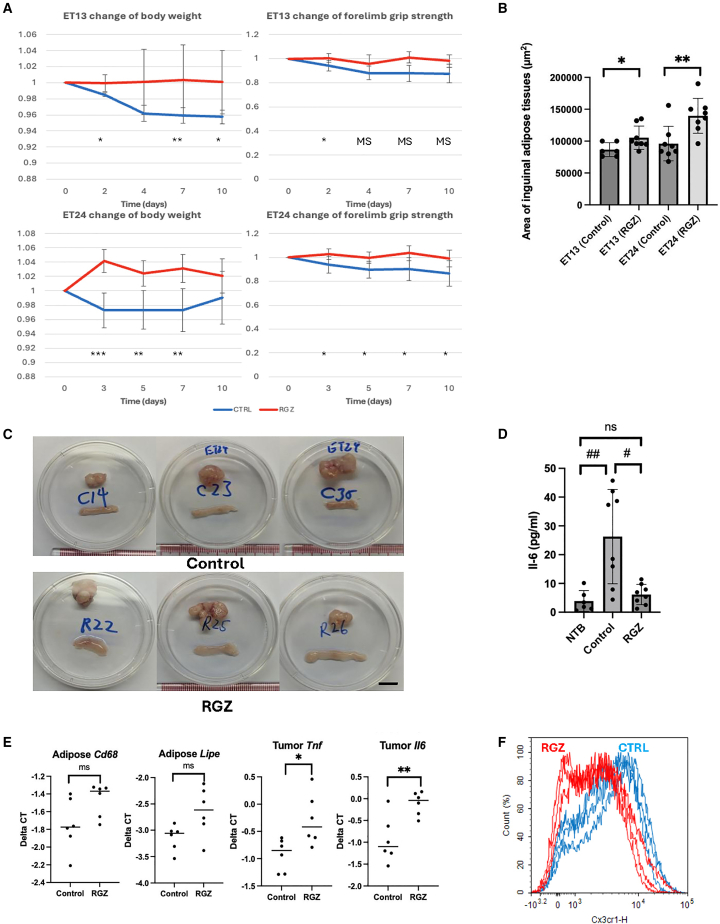
RGZ treatment effectively attenuates cachexia in ESCC PDOX models (A) Therapeutic effects of RGZ on body weight and forelimb grip strength in ET13 and ET24 PDOX-bearing mice, comparing RGZ-treated (red) and solvent-treated control mice (blue). (B) Quantitative analysis of inguinal adipose tissue sizes in PDOX-bearing mice following RGZ treatment. (ET13 (control) = 86667, 95% CI [77921–95412]; ET13 (RGZ) = 105375, 95% CI [92889–117861]; ET24 (Control) = 96125, 95% CI [76027–116223]; ET24 (RGZ) = 139750, 95% CI [123572–155928]). (C) Representative images demonstrating reduced adipose tissue wasting in RGZ-treated ET24 PDOX-bearing mice compared with controls. Images were selected to illustrate the representative phenotype, excluding extreme outliers. *Scale bars, 10 mm*. (D) Plasma IL-6 cytokine levels quantified by ELISA, showing reductions in RGZ-treated mice. (NTB = 25.52, 95% CI [13.41–37.62]; control = 108.14, 95% CI [45.96–170.32]; RGZ = 38.18, 95% CI [30.53–45.82]). (E) RT-qPCR analysis of bulk tumor and organ samples, demonstrating reduced expression of cachexia-related genes *Cd68* and *Lipe* in adipose tissue and *Il6* and *Tnf* in tumors following RGZ treatment. Delta CT normalized to Tbp (higher delta CT indicates reduced expression). (F) Flow cytometric quantification of inflammatory macrophages (Cx3cr1-high) in RGZ-treated versus control mice, showing reduced macrophage populations post-treatment.

Daily oral administration of RGZ (10 mg/kg) significantly attenuated body weight loss, muscle atrophy, and adipose tissue wasting without impacting tumor growth. This dosage has previously been reported as well tolerated and non-toxic in mice.^21^ Compared with vehicle-treated controls, RGZ-treated mice exhibited significant improvements in body weight (+3.13 ± 1.95% vs. −2.66 ± 2.99%; *p* = 0.002), forelimb grip strength (+3.56 ± 6.26% vs. −9.78 ± 9.58%; *p* = 0.024), and adipose tissue retention at the endpoint (139,750 ± 18,907 μm^2^ vs. 96,125 ± 18,779 μm^2^; *p* = 0.006) (Figures 2A–2C and S2). Correspondingly, systemic inflammation was significantly reduced, as indicated by decreased levels of TNFα and IL-6 (Figures 2D and 2E).

Surprisingly, flow cytometry and reverse-transcription-qPCR analyses revealed a pronounced global depletion of pro-inflammatory macrophages upon RGZ treatment; in the adipose tissues, there was down-regulation of key lipolytic genes alongside depleted macrophages (Figures 2E and 2F).^22^ The parallel reduction in macrophage abundance, cytokine levels, and lipolysis suggests that modulating macrophage-driven inflammation is a central mechanism through which RGZ protects against cachexia. This suggests that directly targeting macrophages provide an effective anti-cachexia strategy.

### Macrophage-targeted therapy using PLX3397 on esophageal cancer cachexia

Motivated by the above findings, we next tested a global macrophage-targeted therapeutic strategy against cancer cachexia. To this end, we orally administered the CSF1R inhibitor PLX3397 (40/mg/kg) across a comprehensive panel of four ESCC PDOX models exhibiting varying cachexia severities, enabling systematic evaluation of macrophage-targeted therapy for cancer-associated cachexia. This dosage of PLX3397 has been widely used in preclinical studies, demonstrating good tolerability and no signs of toxicity. The human equivalent dose of the 40 mg/kg used in our study is approximately 227 mg/day for a 70 kg adult, which falls within the dosing range evaluated in phase I clinical trials, where PLX3397 was shown to be well tolerated.^23^

**Figure 3:**
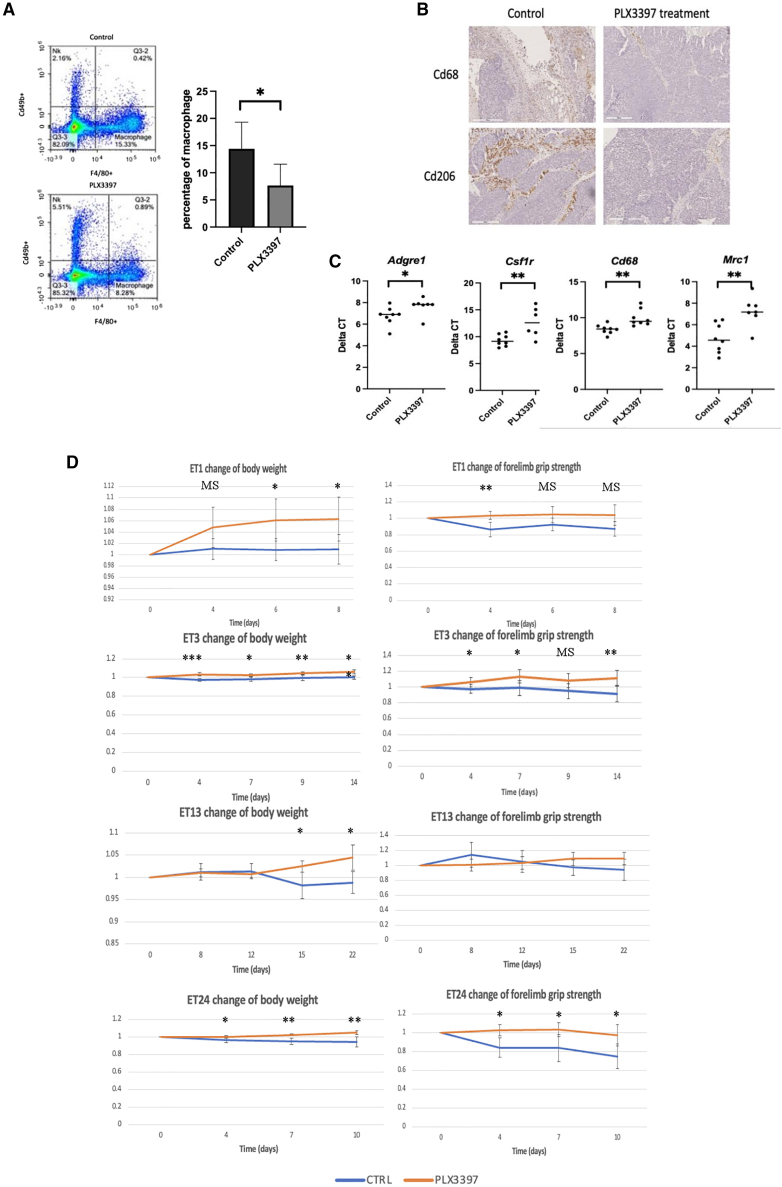
Macrophage-targeted therapy (PLX3397) alleviates cachexia in PDOX models (A) Flow cytometry analysis of dissociated PDOX tumors from solvent-treated control and PLX3397-treated mice, identifying macrophages as H-2Kd+ and F4/80+. The quantitative summary (right) confirms significant macrophage depletion by PLX3397. (B) Immunohistochemistry (IHC) staining for macrophage markers Cd68 and Cd206, illustrating a reduction in total and M2-like macrophages following PLX3397 treatment. (C) RT-qPCR analyses of bulk RNA from tumor and host tissues, confirming decreased expression of macrophage-related markers post-treatment. (D) Functional evaluation of PLX3397’s anti-cachexia efficacy in ET1, ET3, ET13, and ET24 PDOX lines, demonstrating improved body weight and forelimb grip strength in PLX3397-treated mice (orange) relative to controls (blue).

**Figure 4:**
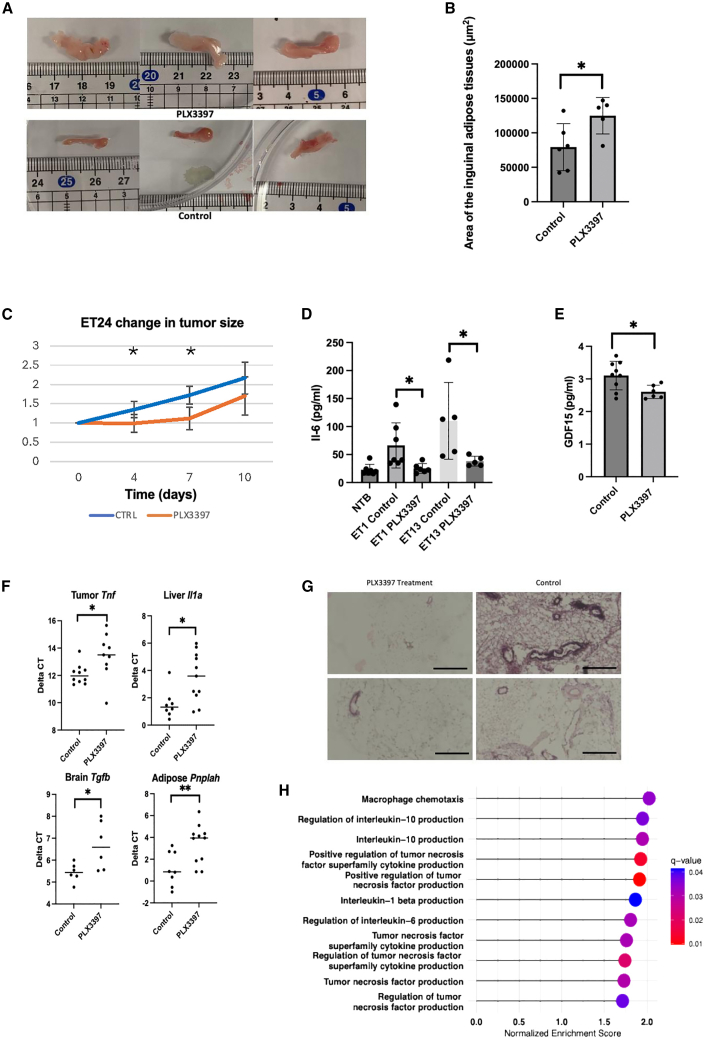
PLX3397 reduces inflammation and preserves adipose tissue integrity in PDOX-bearing mice (A) Representative images of inguinal adipose tissue from ET24-bearing mice, showing reduced wasting in PLX3397-treated mice compared with controls. Images were selected to illustrate the representative phenotype, excluding extreme outliers. (B) Quantitative summary of inguinal adipose tissue sizes after PLX3397 treatment across PDOX models: (control = 79333, 95% CI [52111–106556]; PLX3397 = 124900, 95% CI [101700–148100]). (C) Evaluation of the anti-tumor efficacy of PLX3397, showing minimal effects on tumor size in treated (orange) versus control (blue) groups. (D) Plasma IL-6 cytokine levels measured by ELISA, illustrating reduced systemic inflammation in PLX3397-treated mice (ET1 and ET13): NTB = 22.72, 95% CI [14.82–30.62]; ET1 Control = 66.3, 95% CI [46.58–86.02]; ET1 PLX3397 = 25.22, 95% CI [18.29–32.16]; ET13 Control = 110.14, 95% CI [50.06–170.22]; ET13 PLX3397 = 38.18, 95% CI [30.53–45.82]. (E) Plasma GDF15 cytokine levels (ELISA) significantly reduced following PLX3397 treatment: control = 3.10, 95% CI [2.75–3.45]; PLX3397 = 2.60, 95% CI [2.44–2.77]. (F) RT-qPCR analysis revealing reduced expression of cachexia-related genes *Tnf* in tumor, *Prplah/Atgl* in adipose tissue, *Il1a* and *Il1b* in liver, and *Tgfb* in brain tissues post-PLX3397 treatment, indicative of systemic anti-inflammatory effects. (G) Representative H&E images showing histopathological differences in adipose tissue between PLX3397-treated and control mice. Control mice displayed adipocyte necrosis, marked inflammatory infiltration, and multilocular adipocytes with small lipid droplets, whereas treated mice showed preserved adipose morphology. *Scale bars, 100 μm*. (H) Gene set enrichment analysis (GSEA) using Gene Ontology Biological Process pathways, indicating significant downregulation of macrophage-associated and cachectic cytokine receptor signaling pathways (IL-1β, IL-6, IL-10, TNF) in PLX3397-treated mice relative to controls, suggesting reduced macrophage-driven inflammatory signaling. Results shown as mean ± 95% CI. Statistical significance is labeled as: ###, adjusted *p* value < 0.01, ##, adjusted *p* value < 0.01; #, adjusted *p* value < 0.05; ∗∗∗, *p* value < 0.001; ∗∗, *p* value < 0.01; ∗, *p* value < 0.05; marginally significant (ms), *p* value < 0.1; n.s., not statistically significant.

Effective macrophage depletion, especially the pro-tumor M2-like macrophages, following PLX3397 treatment was confirmed by flow cytometry (Figure 3A), immunohistochemistry staining (Figure 3B), and quantitative PCR analysis (Figure 3C). Notably, PLX3397 treatment consistently led to increased body weight, improved forelimb grip strength, and enlarged IAT across all four PDOX models tested. Specifically, in the representative ET24 PDOX-bearing mice, PLX3397 significantly enhanced body weight (+2.43 ± 1.29% vs. −4.79 ± 3.65% in controls, *p* = 0.004; Figure 3D), grip strength (+3.36 ± 7.48% vs. −16.4 ± 14.4% in controls, *p* = 0.038; Figure 3D), and adipose tissue area (124,900 ± 23,200 μm^2^ vs. 79,333 ± 27,222 μm^2^ in controls, *p* = 0.038; Figures 4A and 4B). Importantly, these benefits occurred with minimal short-term anti-tumor effects (Figure 4C), highlighting macrophage depletion as a promising therapeutic approach for ESCC-associated cachexia.

ELISA assays demonstrated significantly reduced plasma levels of IL-6, a universal cachexia-associated cytokine, in PLX3397-treated mice compared with untreated controls. Levels of human GDF15, another critical cytokine implicated in cachexia, were also notably decreased following PLX3397 treatment (Figures 4D and 4E). Transcriptomic analysis further confirmed these findings, showing global downregulation of cachexia-related genes—such as *Tnf*, *Il1*, and *Tgfb*—in tumor, liver, and brain tissues, respectively, supporting the anti-cachexia efficacy of PLX3397 (Figure 4F). Histopathological examination revealed necrotic adipose tissue with prominent cellular infiltration in untreated cachectic mice, features that were markedly absent in PLX3397-treated animals (Figure 4G). Correspondingly, PLX3397 treatment reduced expression of the adipocyte-associated gene *Pnpla2* (Figure 4F), indicating improved preservation of adipose tissue through inflammatory macrophage depletion. Together, these results suggest that broad macrophage targeting effectively attenuates the systemic inflammation driving cachexia. Supporting this, gene set enrichment analysis (GSEA) revealed significant downregulation of receptor pathways for key cachectic cytokines, including IL-1β, IL-6, IL-10, TNF, and a relative hypersensitivity gene set (Figures 4H and Table S3).

## Discussion

Cancer cachexia is a chronic, progressive syndrome, with metabolic and inflammatory alterations preceding the onset of overt clinical symptoms.^24^ In ESCC, one of the most cachexia-associated cancers with a high incidence rate, cancer cachexia remains largely underexplored, with no patient-derived models available for mechanistic or therapeutic studies.^1^^,^^2^^,^^25^ Conventional CDX models fail to capture this process due to rapid tumor growth that leads to early humane endpoints, preventing sustained tumor-host interactions. In contrast, our PDOX model exhibits slower growth kinetics, enabling the development of key metabolic and inflammatory features associated with cachexia. Notably, we observed variability in cachexia severity across PDOX-bearing mice, even in genetically identical immunodeficient hosts; this reflects the heterogeneous nature of the syndrome, shaped by tumor-intrinsic and patient-specific factors. Our PDOX model mirrors the clinical heterogeneity of cachexia, offering a versatile platform for investigating cachexia mechanisms and interventions. It represents a substantial advance over traditional xenografts and supports more reliable preclinical testing.^25^

Orthotopic cancer models have been proposed as more physiologically relevant systems; however, mice inoculated with esophageal orthotopic xenografts rarely survive beyond 2–3 weeks in mice, limiting their utility for studying chronic conditions such as cachexia or for drug evaluation.^26^ ESCC-associated cachexia has traditionally been attributed to mechanical obstruction of the esophagus, impairing nutrient intake.^27^^,^^28^ However, our PDOX model—free from such anatomical constraints—highlights the substantial contribution of systemic inflammation to ESCC cachexia.

RGZ, a PPAR-γ activator initially developed as an anti-diabetic drug for enhancing insulin sensitivity and lipid metabolism, was selected as another candidate due to its established role in these processes.^29^ Our study shows that RGZ holds promise as an anti-cachexia agent in ESCC. PPAR-γ is highly expressed in macrophages and influences macrophage polarization, thereby reducing their inflammatory state and suppressing key cachectic cytokines.^30^ Asp et al. demonstrated that RGZ can alleviate cancer cachexia by decreasing pro-inflammatory macrophage populations.^19^ Our findings support this, indicating that RGZ prevents cancer cachexia phenotypes by reducing pro-inflammatory cytokine secretion, particularly in adipose tissue. Additionally, studies suggest that PPAR-γ, which is highly expressed in adipose tissue, can promote adipocyte differentiation and lipogenesis, potentially preventing lipid accumulation and insulin resistance, both of which are associated with cancer cachexia.^19^^,^^31^^,^^32^

Interestingly, pexidartinib/PLX3397, a macrophage-targeted CSF1R inhibitor currently in several anti-cancer clinical trials,^33^^,^^34^ shows promise as an anti-cachexia treatment in our study. Macrophages play a key role in cytokine secretion and inflammation.^35^^,^^36^ We observed that depleting macrophages reduced cachectic cytokines Il-1, Il-6, and Tnf-α. Previous studies have shown that pro-tumor M2-like macrophages stimulate lipolysis via noradrenaline production in highly inflamed tumor microenvironments,^37^^,^^38^^,^^39^ suggesting that targeting these cells could alleviate cachexia. Our findings also showed that muscle function was preserved by depleting M2-like macrophages, consistent with reports that these cells contribute to muscle atrophy through the CCL5/TRAF6/NF-κB pathway.^15^^,^^18^ Furthermore, PLX3397 may reduce GDF15—a cachexia-related cytokine—by downregulating TGF-β signaling.^40^^,^^41^^,^^42^^,^^43^^,^^44^ These results suggest that pexidartinib could be an innovative therapeutic option for managing ESCC-associated cachexia.

While not a comprehensive mechanistic dissection, our findings suggest that targeting inflammatory macrophages can effectively mitigate cancer-induced cachexia by restoring metabolic and inflammatory balance. Liu et al. demonstrated in pancreatic cancer that interrupting tumor-macrophage crosstalk can ameliorate cachexia^15^; our findings extend this concept to esophageal cancer and to global inflammation, suggesting a potentially generalizable therapeutic principle for cancer cachexia. Our model replicates clinical cachexia phenotypes and supports RGZ and pexidartinib/PLX3397 as candidate therapeutic. Modulating macrophage activity emerges as a much-needed, promising therapeutic approach for combating cachexia, warranting further investigation into macrophage subsets and potential combination therapies in future anti-cachexia treatments.

### Limitations of the study

Several limitations of our study should be noted. First, our PDOX system is established in immunodeficient nude mice, which precludes analysis of adaptive immune contributions to cachexia. Second, cachexia development showed inter-individual variability even in genetically identical hosts, mirroring the heterogeneity seen in patients but complicating experimental consistency. Third, the 10-day drug treatment was designed as a proof-of-concept rather than a chronic regimen, and extending treatment was not feasible because 20%–30% of untreated animals reached humane endpoints (≥15% body weight loss with functional decline) within 2 weeks, thereby limiting sample size. Fourth, because bulk RNA sequencing (RNA-seq) was performed only on tumor tissue, it does not capture cross-species interactions with host organs such as muscle or adipose tissue. Finally, while our model highlights inflammation-driven cachexia, other mechanisms—including neuroendocrine or tumor-derived catabolic factors—remain unexplored. Despite these limitations, our PDOX model represents a significant advance, as it recapitulates clinically relevant cachexia phenotypes and provides a platform to test anti-cachexia therapies and future combination strategies with standard ESCC treatments.

## Resource availability

### Lead contact

Further information and requests for resources, reagents, or data should be directed to the lead contact, Valen Zhuoyou Yu (email: zvyu@hku.hk, The University of Hong Kong). Dr. Yu is responsible for material requests and will facilitate distribution of unique resources reported in this study.

### Materials availability

All unique materials generated in this study are available from the lead contact. This includes the PDO lines and PDOX tumor samples established for esophageal cancer cachexia research. Organoid lines will be shared in compliance with institutional guidelines and patient consent provisions. No new cell lines requiring additional validation were generated beyond the organoids described. There are no restrictions on reagent availability beyond standard agreements.

### Data and code availability

• Data reported in this study will be shared by the lead contact upon request.
• This study does not contain original code.
• Any additional information required to reanalyze the data reported in this study is available from the lead contact.

## Acknowledgments

We thank the Hong Kong Special Administrative Region Research Grants Council (TRS T12-701/17-R to M.L.L. and S.L.) and the Health Bureau (HMRF 06171566 to V.Z.Y.) for funding support. We thank the Leibniz Institute DSMZ - German Collection of Microorganisms and Cell Cultures GmbH for providing the KYSE cell lines. We thank the University of Hong Kong Li Ka Shing Faculty of Medicine Center for Comparative Medicine Research for animal facilities. We also thank the University of Hong Kong Li Ka Shing Faculty of Medicine Center for PanorOmic Sciences for sequencing and imaging facilities.

## Author contributions

Conceptualization, B.C.L. and V.Z.Y.; methodology, B.C.L., A.K.L., and V.Z.Y.; investigation, B.C.L., A.K.L., C.W.-Y.W., I.Y.W., C.C.H.L., A.W.L., J.M.-Y.K., W.D., D.L.K., S.L., and V.Z.Y.; visualization, B.C.L. and A.K.L; funding acquisition, S.L., M.L.L., and V.Z.Y.; keywords: project administration, M.L.L. and V.Z.Y.; supervision, V.Z.Y.; writing – original draft, B.C.L. and A.K.L.; writing – review & editing, M.L.L. and V.Z.Y.

## Declaration of interests

All authors declared no conflict of interest.

## STAR★Methods

### Key resources table

**Table:** 

REAGENT or RESOURCE	SOURCE	IDENTIFIER
**Antibodies**		

CD68 (Rabbit monoclonal)	Cell Signaling Technology	Cat# 97778; RRID: AB_2928056
CD206 (Rabbit monoclonal)	Cell Signaling Technology	Cat# 24595; RRID: AB_2892682
H-2Kd (Mouse monoclonal, clone SF1-1.1)	BioLegend	Cat# 116628; RRID: AB_2616846
F4/80 (Rat monoclonal, clone BM8)	BioLegend	Cat# 123114; RRID: AB_893478
CX3CR1 (Mouse monoclonal)	BioLegend	Cat# 149013; RRID: AB_2565697

**Chemicals, peptides, and recombinant proteins**		

Rosiglitazone (RGZ, BRL-49653)	MedChemExpress	Cat# HY-17386
Pexidartinib (PLX-3397)	MedChemExpress	Cat# HY-16749
Matrigel Matrix (Growth Factor Reduced)	Corning	Cat# 354230

**Critical commercial assays**		

Mouse IL-6 ELISA Kit	Proteintech	Cat# KE10007
Human GDF-15 ELISA Kit	Proteintech	Cat# KE00108

**Deposited data**		

Bulk RNA-seq data (this study)	NCBI BioProject	BioProject ID PRJNA1274150

**Experimental models: Cell lines**		

KYSE-70 (human ESCC cell line)	DSMZ (original provider: JCRB)	Cat# ACC-363; RRID: CVCL_1356
KYSE-150 (human ESCC cell line)	DSMZ (original provider: JCRB)	Cat# ACC-375; RRID: CVCL_1348
KYSE-180 (human ESCC cell line)	DSMZ (original provider: JCRB)	Cat# ACC-379; RRID: CVCL_1349
KYSE-450 (human ESCC cell line)	DSMZ (original provider: JCRB)	Cat# ACC-387; RRID: CVCL_1353

**Experimental models: Organisms/strains**		

Mouse: BALB/cAnN-Foxn1 nu (nude, immunodeficient)	HKU Center for Comparative Medicine Research	–
Mouse: C.B-17/Icr-Prkdc scid (SCID, immunodeficient)	HKU Center for Comparative Medicine Research	–

**Oligonucleotides**		

Primers for quantitative PCR (mouse Il6, Tnf, Cd68, etc.; see Table S2)	This paper	–

**Software and algorithms**		

Partek Flow (NGS data analysis software)	Partek Inc.	https://www.partek.com/products/flow/
clusterProfiler (v4.3.3, gene set enrichment analysis package in R)	Bioconductor	RRID: SCR_016884
R (statistical computing environment), version 4.3.3	R Foundation for Statistical Computing	https://www.r-project.org

### Experimental model and study participant details

#### Human tissue samples and patient-derived organoids

Primary esophageal squamous cell carcinoma (ESCC) specimens were obtained from patients at Queen Mary Hospital (Hong Kong) with informed consent, under protocols approved by the Institutional Review Board (The University of Hong Kong/Hospital Authority Hong Kong West Cluster). Each tumor sample was collected either endoscopically at diagnosis or during surgical resection. Patients’ clinical details (age, sex, tumor stage, treatment history) corresponding to each PDO line are summarized in Yu et al. (2024) and Table S1.^18^

The generate and passage of the patient-derived organoids (PDOs) were detailly listed in Yu et al.^18^

#### Mice and animal husbandry

All animal experiments were approved by the Committee on the Use of Live Animals in Teaching and Research (CULATR) of The University of Hong Kong, and conducted in accordance with institutional and AAALAC guidelines. Female immunodeficient mice were used for xenograft studies: specifically, BALB/cAnN-nu nude mice and C.B-17/Icr-scid SCID mice (6–10 weeks old, ∼18–22 g). These mice were chosen for their lack of T cell (nude) or both T- and B-cell (SCID) immunity, enabling engraftment of human cells. Mice were obtained from the HKU Center for Comparative Medicine Research (CCMR) breeding colony and housed in individually ventilated cages under specific pathogen-free conditions. The vivarium was maintained at 26 °C ambient temperature with a 12-h light/dark cycle. Animals had free access to standard chow diet and water *ad libitum*, and were allowed to acclimate for at least one week before any procedures. For this study, a total of ∼100 mice were used, with cohort sizes detailed below for each experiment. Non-tumor-bearing (NTB) control mice (age-matched and strain-matched) were included as negative controls for cachexia phenotypes.

All efforts were made to minimize animal suffering: anesthesia (ketamine/xylazine) was used for surgical procedures, and analgesics (buprenorphine and meloxicam) were provided post-operatively as needed. Humane endpoints (maximum tumor size ∼1.5 cm diameter, severe lethargy, or >20% body weight loss) were predefined in the animal protocol, and mice were euthanized by pentobarbital overdose or CO_2_ inhalation at endpoint.

#### Cell lines for comparison (CDX models)

In addition to PDOX models, conventional cell line-derived xenografts (CDXs) were used for baseline comparisons of tumor growth and cachexia (as referenced in the text). The human ESCC cell lines KYSE70, KYSE150, KYSE180, and KYSE450 were originally obtained from JCRB/DSMZ and cultured in RPMI-1640 medium +10% FBS. While the main focus is on PDOX, these cell lines were used in a limited manner to establish fast-growing xenografts and orthotopic tumors for comparative purposes (see method details below). All cell lines were authenticated and tested mycoplasma-free. Details of cell culture was as previously described.^15^

### Method details

#### Establishment of PDOX models

For PDOX generation, we expanded each PDO line *in vitro* and then inoculated organoid-derived cells into mice. Organoids designated for xenografting were collected from culture, and enzymatically dissociated to single cells (using TrypLE or the same dissociation buffer described above). Typically, a confluent 24-well of organoids yielded ∼1–2 × 10^6^ cells for injection. Cell suspensions were mixed 1:1 with Matrigel (50 μL total per injection) to promote cell engraftment. SCID mice were used for initial engraftment because of their higher take rate for human tissues. Mice (7–8 weeks old) were anesthetized with isoflurane and given a single subcutaneous injection of ∼0.5 × 10ˆ6 organoid-derived cells in the flank (unilateral injection). In the first passage (establishment phase), SCID mice were used to allow robust tumor take. Tumor growth was monitored by caliper measurements twice weekly. Once a PDOX tumor reached ∼150–300 mm^3^ or if the mouse exhibited cachexia symptoms, the animal was humanely sacrificed and the tumor harvested. To maintain and expand the PDOX lines, each harvested tumor was dissected and dissociated into single cells or small clusters using the same collagenase-based protocol as for patient tissues previously described in Yu et al.^18^ The dissociated PDOX cells were either cryopreserved or reinjected into new recipient mice (usually SCID or nude mice) for propagation (plain RPMI medium instead of matrigel was used). Subsequent passages (P1, P2, etc.) were done in nude mice to facilitate downstream immunological assays (nude mice are T cell deficient but have functional innate immune cells, including macrophages). For each PDOX line, we performed at least one passage *in vivo* to ensure a stable and expandable line. The overall take rate for PDOX establishment was ∼40%, consistent with other organoid xenograft studies.

#### Conventional xenograft (CDX) generation

For comparison of growth kinetics and cachexia, we also generated xenografts from ESCC cell lines. Details of CDX generation was as previously described.^18^

#### Cachexia phenotype monitoring

Tumor-bearing mice (PDOX or CDX) and control NTB mice were monitored at least 1–2 times weekly for cachexia-related parameters. These included body weight, food intake, and skeletal muscle strength, as detailed below.

• Body Weight: Each mouse was weighed on an electronic scale, and weight was recorded from the day of tumor inoculation (baseline) through the course of the experiment. Weight change is presented as a percentage of the baseline weight. In cachexia assessments, a significant difference in body weight/muscle function changes comparing to the NTB healthy mice (from the same mice batch) was considered onset of cachexia. Food intake was measured per cage; importantly, food consumption remained similar between tumor-bearing and control groups, indicating that anorexia was not a major factor and weight loss was due to metabolic wasting.
• Grip Strength: Forelimb muscle strength was measured using a digital grip strength meter (BioSeb, Chaville, France). Each mouse was allowed to grasp a metal grid with its forepaws, then gently pulled backward until it released; the peak force (in grams-force, gf) was recorded. We took 5 measurements per session and took the highest reading for each time point. Baseline grip strength was measured prior to tumor implantation, then monitored weekly. A decline in grip strength is a functional readout of skeletal muscle weakness/atrophy.
• Adipose and Muscle Wasting Measurements: At sacrifice, we dissected the bilateral inguinal white adipose tissue (IAT) pads from each mouse. These tissues were immediately either frozen or fixed for histology. We quantified adipose tissue area histologically as another measure of fat wasting.

All measurements above were included in our analysis for every individual mouse. Mice that died or reached endpoint early were included up until their last observation to avoid bias. NTB control mice (housed under identical conditions) maintained stable body weight and grip strength, serving as a baseline reference.

#### Pharmacological treatments

We evaluated two candidate anti-cachexia agents in the PDOX model: Rosiglitazone (RGZ) and Pexidartinib (PLX3397). These drugs were chosen based on their known effects on inflammation and metabolism (PPARγ activation^20^ and CSF1R inhibition,^22^ respectively). Below are the treatment protocols.

• Rosiglitazone (RGZ): RGZ (MedChemExpress) was formulated fresh on the day of treatment in a vehicle of 5% DMSO, 45% PEG-300, 5% Tween-80, 45% sterile saline. Mice received RGZ at 10 mg/kg per day via oral gavage. Treatment was initiated once tumors were established and cachexia signs began to manifest (and tumor volume ≥150 mm^3^), then started daily RGZ dosing. The treatment duration was 10 days on average, after which control mice typically approached humane endpoints (due to cachexia severity or tumor size). Mice in the control group received vehicle gavage on the same schedule. RGZ dosing at 10 mg/kg was based on literature showing efficacy in murine cachexia models and was well-tolerated (no overt toxicity was seen; treated mice remained active).^21^
• Pexidartinib (PLX3397): PLX3397 (MedChemExpress) was prepared in the same vehicle as above. Mice were treated with PLX3397 at 40 mg/kg per day by oral gavage. The timing of treatment start was similar to RGZ: when cachexia became evident. PLX3397 was given for 10 consecutive days, or until the control mice reached endpoint. This dosing and schedule were guided by prior studies demonstrating effective macrophage depletion *in vivo* at ∼30–50 mg/kg daily. All mice were observed daily for signs of drug toxicity; none showed significant weight loss beyond the tumor-induced cachexia or other adverse effects attributable to the drugs. Importantly, food intake remained stable during treatments, indicating the drugs did not cause anorexia.
• Randomization and Group Size: For each drug test, mice with established PDOX tumors were randomly assigned to treatment vs. vehicle groups (using a random number method) once they met the inclusion criterion (cachexia onset). Group sizes were 6–9 mice per group for each treatment, as specified in our study design. This sample size provided adequate power (>80%) to detect meaningful improvements in weight loss or muscle mass, based on power calculations (assuming ∼30% effect size). We also ensured that each PDOX line used for treatment had representation in both control and treated groups to account for any line-specific differences. No blinding was done during treatment due to the nature of drug administration, but outcome assessments (like histology grading and flow cytometry analysis) were performed blinded to treatment group.
• Treatment Endpoints: The primary endpoints for treatment efficacy were body weight change, grip strength, adipose tissue mass, and circulating cytokine levels at the end of the treatment period. Mice were euthanized when the vehicle group reached humane endpoint criteria (typically ∼10 days of treatment for cachexia experiments). At that time, all mice were sacrificed and tissues collected for analysis (to allow fair comparison between groups). Tumor sizes at endpoint were also recorded; notably, neither RGZ nor PLX3397 had a strong effect on tumor growth over this short period (as expected, since they are not cytotoxic drugs). However, both agents had significant effects on cachexia metrics, as reported in the Results.

#### Histology and immunohistochemistry

Tissues collected at necropsy (tumors, muscle, fat, liver, etc.) were fixed in 10% neutral-buffered formalin for 16–24 h and then processed to paraffin blocks. Paraffin-embedded samples were cut into 4–5 μm sections and mounted on slides. Hematoxylin and eosin (H&E) staining was performed on sections of tumor and tissues for morphological evaluation. All PDOX tumor sections were reviewed by a pathologist to confirm ESCC morphology. We also examined adipose tissue sections (IAT).

For immunohistochemistry (IHC), sections underwent deparaffinization and antigen retrieval (citrate buffer pH 6.0, 95 °C) and were stained with specific primary antibodies to identify macrophages in tissues.

#### Flow cytometry (immune cell analysis)

We utilized flow cytometry to further characterize the immune cell populations in PDOX tumors, particularly to verify the effect of PLX3397 on tumor-associated macrophages (TAMs). Single-cell suspensions were prepared from fresh tumor tissues by mechanical dissociation and enzymatic digestion as previously described.^18^

#### Bulk RNA sequencing and gene expression analysis

Bulk transcriptomic analysis was performed as previously described.^18^ In brief, poly-A enriched mRNA sequencing of PDOX samples treated with PLX3397 or solvent controls was performed at the HKUMed Center for PanorOmic Sciences. Sequencing reads were aligned to human and mouse transcriptomes, and data analysis was conducted using Partek Flow software. GSEA was performed using the clusterProfiler package in R (version 4.3.3), employing pathways from the Gene Ontology Biological Process (GOBP) database, with significance set at *p* < 0.05. The transcriptomic data are available (BioProject ID PRJNA1274150).

#### Bulk RNA sequencing and gene set enrichment analysis (GSEA)

Bulk transcriptomic analysis was performed as previously described.^18^ In brief, poly-A enriched mRNA sequencing of PDOX samples treated with PLX3397 or solvent controls was performed at the HKUMed Center for PanorOmic Sciences. Sequencing reads were aligned to human and mouse transcriptomes, and data analysis was conducted using Partek Flow software. GSEA was performed using the clusterProfiler package in R (version 4.3.3), employing pathways from the Gene Ontology Biological Process (GOBP) database, with significance set at *p* < 0.05. The transcriptomic data are available (BioProject ID PRJNA1274150).

#### Quantitative PCR validation

To validate key transcriptomic findings, we performed RT-qPCR on select targets. Total RNA was isolated from RNAlater-stored tissues using TRIzol reagent (Thermo Fisher Scientific). cDNA synthesis was performed with 0.2 μg RNA using the QuantiNova Reverse Transcription Kit. RT-qPCR was conducted using a Roche LightCycler480 II system, and qPCR was run for various macrophage or cachexic related genes and housekeeping gene *Tbp*, with primer sequences detaily listed in Table S2.

#### Cytokine measurements (ELISA)

Systemic cytokine levels were measured to link molecular changes to cachexia phenotypes. Blood was collected via cardiac puncture at sacrifice and plasma separated by centrifugation. We used enzyme-linked immunosorbent assay (ELISA) kits to quantify pro-inflammatory cytokines. Plasma levels were quantified according to the manufacturer’s instructions.

### Quantification and statistical analysis

All data were derived from at least three independent experiments with a minimum of five animals per group. The data presented includes all mice within the respective groups, accounting for outlier tumor sizes. Statistical significance was assessed using two-sided independent samples t-tests, unless otherwise stated. The adjusted *p*-values were calculated to compute the statistical significance of multiple comparisons employing the Benjamini-Hochberg correction method. Exact *p*-values are listed in Table S4.
