# Supplementary material for: Patient-derived organoid xenografts model esophageal cancer cachexia and enable assessment of anti-inflammatory drug repositioning
Source: iScience. 2026 Jan 7;29(2):114638. doi: 10.1016/j.isci.2026.114638 (PMC12859199; doi:10.1016/j.isci.2026.114638)
Supplement: Document S1. Figures S1 and S2 and Tables S1–S4 [file mmc1.pdf]

iScience, Volume *29*

Supplemental information

Patient-derived organoid xenografts model esophageal cancer cachexia and enable assessment of anti-inflammatory drug repositioning

Bryan Chee-chad Lung, Alvin Ka-kiu Leung, Carissa Wing-Yan Wong, Ian Yu-hong Wong, Cheryl Chee Heng Lung, Anthony Wing-ip Lo, Josephine Mun-Yee Ko, Wei Dai, Dora Lai-wan Kwong, Simon Law, Maria Li Lung, and Valen Zhuoyou Yu

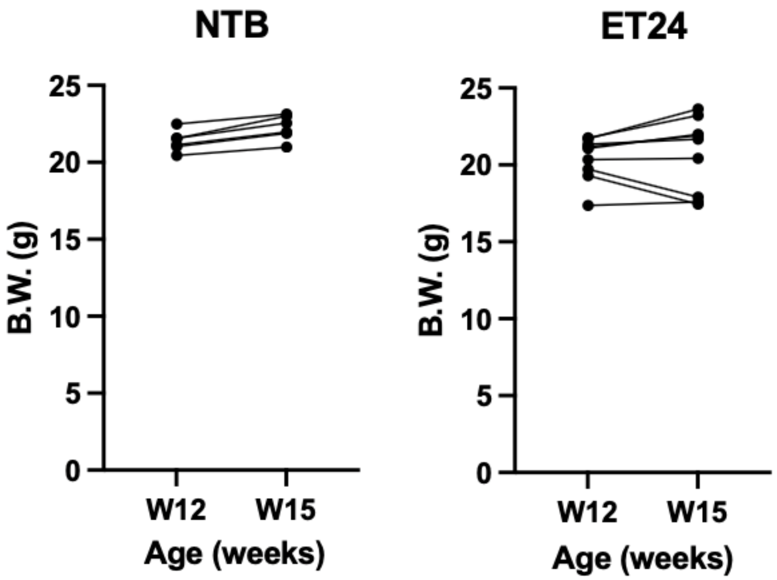

Supplementary Figure 1. Body weight changes for 3 weeks of NTB healthy mice (left), and ET24 bearing mice (mice); stabilized body weight gain showed by the NTB mice (ranged from 2.8% to 6.6% gain), whereas

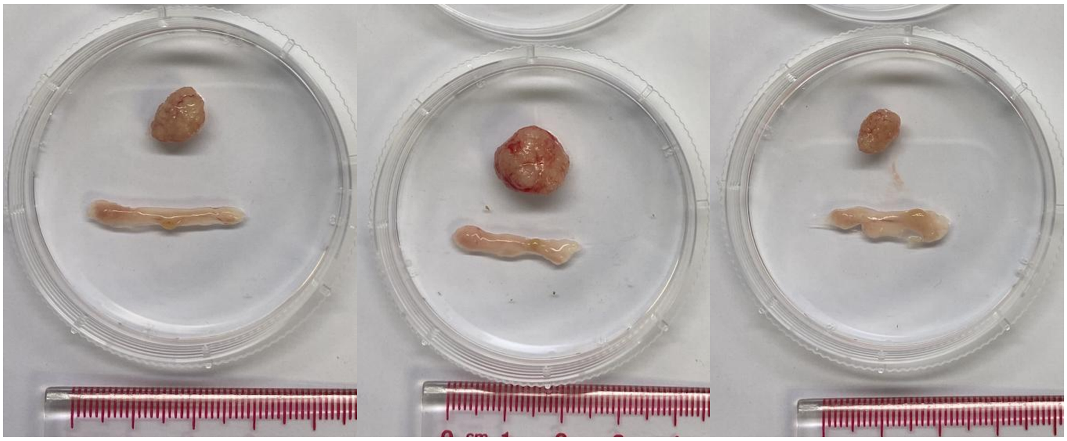

Control

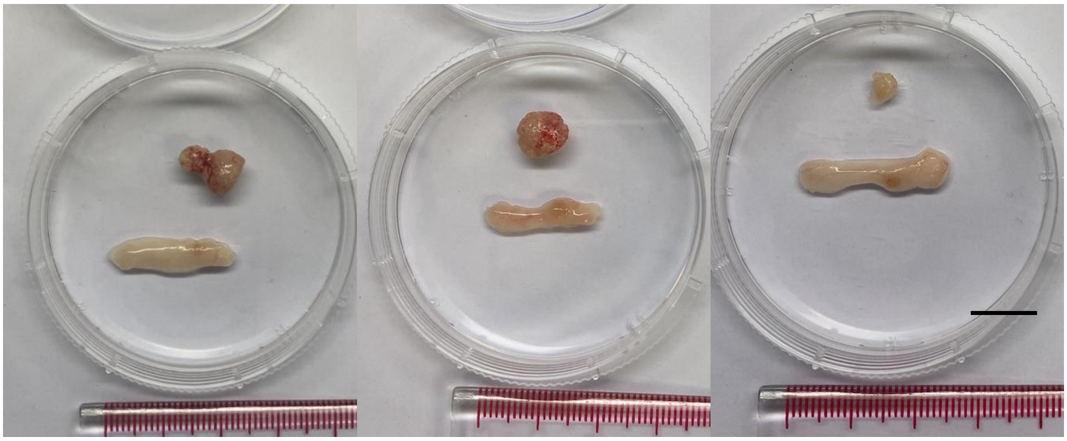

RGZ

Supplementary Figure 2. Representative images demonstrating reduced adipose tissue wasting in RGZ-treated ET13 PDOX-bearing mice compared to controls. *Scale bar = 10 mm*

Supplementary Table 1

PDOs used in this study (apart from ET28 and ET31) were detaily listed in Yu etal. (2024) (18).

Name	*Established organoid type*	*Patient tissue type*	*Sample acquisition method*	*Prior treatment*	*Stage*	*Turnaround time*
ET28	ESCC PDO	Tumor tissue	Endoscopic examination	None	Unknown	1.5 months
ET31	ESCC PDO	Tumor tissue	Endoscopic examination	None	Unknown	2 months

Supplementary Table 2

Primers used in the study.

Target	*Forward oligo*	*Reverse oligo*	*Application*
Il6	GTCTGTAGCTCATTCTGCTCTG	GAAGGCAACTGGATGGAAGT	*Quantitative PCR*
Tnf	CTACCTTGTTGCCTCCTCTTT	GAGCAGAGGTTCAGTGATGTAG	*Quantitative PCR*
Cd68	GACCTACATCAGAGCCCGAGT	CGCCATGAATGTCCACTG	*Quantitative PCR*
Lipe	CATCAACCACTGTGAGGGTAAG	AAGGGAGGTGAGATGGTAACT	*Quantitative PCR*
Adgre1	CGTCAGGTACGGGATGAATATAAG	ATCTTGGAAGTGGATGGCATAG	*Quantitative PCR*
Csf1r	CTGGGACAGCACGAGAATATAG	CCTTCGGAGAAAGTTGAGTAGG	*Quantitative PCR*
Mrc1	CCACAGCATTGAGGAGTTTG	ACAGCTCATCATTTGGCTCA	*Quantitative PCR*
Il1a	GCTTGAGTCGGCAAAGAAATC	GAGAGATGGTCAATGGCAGAA	*Quantitative PCR*
Il1b	ATGGGCAACCACTTACCTATTT	GTTCTAGAGAGTGCTGCCTAATG	*Quantitative PCR*
Tgfb	GGTGGTATACTGAGACACCTTG	CCCAAGGAAAGGTAGGTGATAG	*Quantitative PCR*
Pnplah	TAGCTAACAGTTGGGCTTCAC	CAGAGAGAACAGAGCAGCTTAC	*Quantitative PCR*
Tbp	GGGGAGCTGTGATGTGAAGT	CCAGGAAATAATTCTGGCTCAT	*Quantitative PCR*
RPS13	GGTTGAAGTTGACATCTGACGA	CTTGTGCAACACCATGTGAAT	*Quantitative PCR*

Supplementary Table 3

Gene set enrichment analysis (GSEA) was conducted utilizing the clusterProfiler package (version 4.10.1) within the R statistical environment (version 4.3.3) to ascertain potential concordant variances between the solvent treated control group and the PLX3397 treated group (BioProject ID PRJNA1274150).

ID	Description	setSize	enrichmentScore	NES	pvalue	p.adjust	qvalue
GO:0048246	macrophage chemotaxis	37	0.680233834	2.024068	0.00128115	0.0375279	0.03426781
GO:0032653	regulation of interleukin-10 production	41	0.650694048	1.947263	0.0013971	0.0398442	0.03638287
GO:0032613	interleukin-10 production	44	0.640780829	1.945845	0.00109684	0.0338979	0.03095309
GO:1903557	tumor necrosis factor superfamily cytokine production	110	0.546879853	1.924539	0.00033316	0.0143005	0.01305817
GO:0032760	tumor necrosis factor production	109	0.54347191	1.911484	0.00022321	0.0107139	0.00978315
GO:0032611	production	75	0.562614479	1.863203	0.00169225	0.0451688	0.04124491
GO:0032675	production	132	0.502430649	1.806707	0.00117281	0.0347103	0.03169495
GO:0071706	superfamily cytokine production	174	0.473073896	1.758668	0.00114439	0.0342237	0.03125065
GO:1903555	necrosis factor superfamily cytokine	168	0.46990289	1.740401	0.00061016	0.0232347	0.02121627
GO:0032640	production	172	0.466458836	1.729433	0.00103826	0.0329474	0.03008524
GO:0032680	regulation of tumor necrosis factor production	166	0.463034401	1.712383	0.00149585	0.0414771	0.0378739
GO:0002524	hypersensitivity	26	0.714860067	1.987522	0.00106987	0.0337628	0.03082981

Supplementary Table 4

Exact p-values of statistical significance caculations in figures.

Figure	Statistical test	Comparison	Adjusted p / p-value	symbol
1E	two-tailed unpaired t-test	NTB vs ET1	0.031	#
1E	two-tailed unpaired t-test	NTB vs ET5	0.0002	###
1E	two-tailed unpaired t-test	NTB vs ET13	0.002	##
1E	two-tailed unpaired t-test	NTB vs ET24	0.004	##
1F	two-tailed unpaired t-test	NTB vs ET24 (Week 13)	0.048	*
1F	two-tailed unpaired t-test	NTB vs ET24 (Week 14)	0.003	**
1F	two-tailed unpaired t-test	NTB vs ET13 (Week 17)	0.046	*
1F	two-tailed unpaired t-test	NTB vs ET13 (Week 18)	0.002	**
1G	two-tailed unpaired t-test	NTB vs ET1	0.003	##
1G	two-tailed unpaired t-test	NTB vs ET13	0.01	#
1G	two-tailed unpaired t-test	NTB vs ET24	0.049	#
2A	two-tailed unpaired t-test	ET13 change of body weight (day 2)	0.025	*
2A	two-tailed unpaired t-test	ET13 change of body weight (day 7)	0.006	**
2A	two-tailed unpaired t-test	ET13 change of body weight (day 10)	0.041	*
2A	two-tailed unpaired t-test	ET13 change of forelimb grip strength (day 2)	0.046	*
2A	two-tailed unpaired t-test	ET13 change of forelimb grip strength (day 4)	0.098	ms
2A	two-tailed unpaired t-test	ET13 change of forelimb grip strength (day 7)	0.089	ms
2A	two-tailed unpaired t-test	ET13 change of forelimb grip strength (day 10)	0.066	ms
2A	two-tailed unpaired t-test	ET24 change of body weight (day 3)	0.0001	***
2A	two-tailed unpaired t-test	ET24 change of body weight (day 5)	0.004	**
2A	two-tailed unpaired t-test	ET24 change of body weight (day 7)	0.002	**
2A	two-tailed unpaired t-test	ET24 change of forelimb grip strength (day 3)	0.046	*
2A	two-tailed unpaired t-test	ET24 change of forelimb grip strength (day 5)	0.029	*
2A	two-tailed unpaired t-test	ET24 change of forelimb grip strength (day 7)	0.024	*
2A	two-tailed unpaired t-test	ET24 change of forelimb grip strength (day 10)	0.036	*
2B	two-tailed unpaired t-test	ET13 (control vs RGZ)	0.047	*
2B	two-tailed unpaired t-test	ET24 (control vs RGZ)	0.006	**
2D	two-tailed unpaired t-test	NTB vs control	0.007	##
2D	two-tailed unpaired t-test	Control vs RGZ	0.004	#
2D	two-tailed unpaired t-test	NTB vs RGZ	0.26	ns

2E	two-tailed unpaired t-test	Adipose Cd68 (control vs RGZ)	0.077	ms
2E	two-tailed unpaired t-test	Adipose Lipe (control vs RGZ)	0.072	ms
2E	two-tailed unpaired t-test	Tumor Tnf (control vs RGZ)	0.019	*
2E	two-tailed unpaired t-test	Tumor Il6 (control vs RGZ)	0.006	**
3A	two-tailed unpaired t-test	Control vs PLX3397	0.043	*
3C	two-tailed unpaired t-test	Adgre1 (control vs PLX3397)	0.049	*
3C	two-tailed unpaired t-test	Csf1r (control vs PLX3397)	0.004	**
3C	two-tailed unpaired t-test	Cd68 (control vs PLX3397)	0.004	**
3C	two-tailed unpaired t-test	Mrc1 (control vs PLX3397)	0.004	**
3D	two-tailed unpaired t-test	ET1 change of body weight (day 4)	0.093	ms
3D	two-tailed unpaired t-test	ET1 change of body weight (day 6)	0.036	*
3D	two-tailed unpaired t-test	ET1 change of body weight (day 8)	0.049	*
3D	two-tailed unpaired t-test	ET1 change of forelimb grip strength (day 4)	0.008	**
3D	two-tailed unpaired t-test	ET1 change of forelimb grip strength (day 6)	0.081	ms
3D	two-tailed unpaired t-test	ET1 change of forelimb grip strength (day 8)	0.058	ms
3D	two-tailed unpaired t-test	ET3 change of body weight (day 4)	0.0004	***
3D	two-tailed unpaired t-test	ET3 change of body weight (day 7)	0.021	*
3D	two-tailed unpaired t-test	ET3 change of body weight (day 9)	0.001	**
3D	two-tailed unpaired t-test	ET3 change of body weight (day 14)	0.002	*
3D	two-tailed unpaired t-test	ET3 change of forelimb grip strength (day 4)	0.044	*
3D	two-tailed unpaired t-test	ET3 change of forelimb grip strength (day 7)	0.033	*
3D	two-tailed unpaired t-test	ET3 change of forelimb grip strength (day 9)	0.063	ms
3D	two-tailed unpaired t-test	ET3 change of forelimb grip strength (day 14)	0.01	**
3D	two-tailed unpaired t-test	ET13 change of body weight (day 15)	0.039	*
3D	two-tailed unpaired t-test	ET13 change of body weight (day 22)	0.017	*
3D	two-tailed unpaired t-test	ET1 change of body weight (day 4)	0.037	*
3D	two-tailed unpaired t-test	ET1 change of body weight (day 7)	0.004	**
3D	two-tailed unpaired t-test	ET1 change of body weight (day 10)	0.005	**
3D	two-tailed unpaired t-test	ET24 change of forelimb grip strength (day 4)	0.012	*
3D	two-tailed unpaired t-test	ET24 change of forelimb grip strength (day 7)	0.038	*

3D	two-tailed unpaired t-test	ET24 change of forelimb grip strength (day 10)	0.03	*
4B	two-tailed unpaired t-test	Control vs PLX3397	0.038	*
4C	two-tailed unpaired t-test	ET24 Control vs PLX3397 (day 4)	0.05	*
4C	two-tailed unpaired t-test	ET24 Control vs PLX3397 (day 7)	0.01	*
4D	two-tailed unpaired t-test	ET1 Control vs PLX3397	0.033	*
4D	two-tailed unpaired t-test	ET13 Control vs PLX3397	0.048	*
4E	two-tailed unpaired t-test	Control vs PLX3397	0.023	*
4F	two-tailed unpaired t-test	Tumor Tnf (control vs PLX3397)	0.03	*
4F	two-tailed unpaired t-test	Liver Il1a (control vs PLX3397)	0.013	*
4F	two-tailed unpaired t-test	Brain Tgfb (control vs PLX3397)	0.028	*
4F	two-tailed unpaired t-test	Adipose Pnplah (control vs PLX3397)	0.01	**
